# 维布妥昔单抗治疗CD30阳性淋巴瘤的研究进展

**DOI:** 10.3760/cma.j.issn.0253-2727.2023.01.018

**Published:** 2023-01

**Authors:** 铭慈 蔡, 彭鹏 许, 维莅 赵

**Affiliations:** 上海交通大学医学院附属瑞金医院，国家转化医学研究中心，医学基因组学国家重点实验室，上海血液学研究所，上海 200025 National Research Center for Translational Medicine, State Key Laboratory of Medical Genomics; Shanghai Institute of Hematology, Shanghai Rui Jin Hospital, Shanghai JiaoTong University School of Medicine, Shanghai 200025, China

CD30是一种跨膜蛋白，表达于一部分活化的T淋巴细胞和B淋巴细胞。CD30通过核转录因子kappa B（NK-κB）等不同信号通路发挥作用，最终导致CD30表达上调使细胞获得生存优势。虽然其特异性表达于间变T细胞淋巴瘤（ALCL）和霍奇金淋巴瘤（HL），但在其他淋巴瘤亚型中也有不同程度表达，是潜在的治疗靶点。以CD30为靶点的抗体药物偶联物维布妥昔单抗（BV）能够显著改善CD30阳性淋巴瘤患者的生存，为患者提供新的治疗选择。本文将就BV治疗CD30阳性淋巴瘤的研究进展作一综述。

一、CD30分子及其在淋巴瘤中的表达情况

1982年，Schwab等首次在HL细胞系肿瘤细胞和Reed-Sternberg（RS）细胞上发现这一特异性肿瘤生物标志，并在1986年第三届白细胞分化抗原国际工作组会议上将其命名为CD30。CD30是肿瘤坏死因子受体超家族（TNFRSF）的一员，也称为TNFRSF8或Ki-1抗原，是一个相对分子质量为120×10^3^的Ⅰ型跨膜糖蛋白，以三聚体形式存在并表达于细胞表面[1]。

CD30除了在ALCL及HL中特异性强表达外，在皮肤CD30阳性淋巴细胞增殖性疾病，如皮肤间变T细胞淋巴瘤（CTCL）和淋巴瘤样丘疹病（LyP）中也100％特异性表达。其他病理亚型，如弥漫大B细胞淋巴瘤（DLBCL）、外周T细胞淋巴瘤-非特指型（PTCL-NOS）、血管免疫母细胞性T细胞淋巴瘤（AITL）等也在不同程度上表达CD30[2]–[13]（表1）。与ALCL相比，其他病理亚型肿瘤标本CD30染色较弱，且仅在部分肿瘤细胞中表达。

**表1:** CD30在淋巴瘤中的表达情况

病理亚型	CD30表达率
外周T细胞淋巴瘤	
外周T细胞淋巴瘤-非特指型[2]–[4]	40%～64%
血管免疫母细胞性T细胞淋巴瘤[2]–[4]	43%～76%
结外NK/T细胞淋巴瘤[2]–[6]	31%～80%
蕈样肉芽肿[3]	59%
蕈样肉芽肿伴大细胞转化[3]	100%
肠病相关T细胞淋巴瘤（原Ⅰ型）[3]–[4]	54%～100%
单形性亲上皮性肠道T细胞淋巴瘤（原Ⅱ型）[3]–[4]	0～54%
成人T细胞白血病/淋巴瘤[2]	56%
肝脾T细胞淋巴瘤[2]	0
B细胞淋巴瘤	
弥漫大B细胞淋巴瘤[7]–[10]	24%～50%
原发纵隔大B细胞淋巴瘤[11]–[12]	19%～69%
滤泡性淋巴瘤[13]	32%
伯基特淋巴瘤[10]	0
其他^a^	

注 ^a^霍奇金淋巴瘤、系统性及皮肤间变大T细胞淋巴瘤、淋巴瘤样丘疹病均100％表达CD30

二、维布妥昔单抗的作用机制及适应证

维布妥昔单抗是一种靶向CD30抗体药物偶联物（ADC），CD30抗体部分与抗微管剂单甲基澳瑞他汀E（MMAE）通过蛋白酶可切割的连接桥连接。CD30单抗部分与肿瘤细胞表面CD30特异性结合后CD30复合物被细胞快速内吞、转运至溶酶体，连接桥被降解，MMAE被释放到细胞内部，与胞内微管蛋白结合并破坏微管网络，使细胞有丝分裂停滞在G_2_/M细胞周期，从而介导细胞凋亡。另外，MMAE能从CD30阳性淋巴瘤细胞扩散到周围肿瘤微环境中，发挥旁观者效应进行抗肿瘤作用。这也解释了当淋巴瘤细胞CD30表达率非常低（如3％）时，有时BV治疗仍然有效[14]。另外，Lobastova等[15]提出，CD30阳性胞外囊泡（EVs）可能在其中发挥重要作用，研究发现CD30阳性EVs既可以与DLBCL细胞株结合，也能够和荧光素标记的BV结合，从而使BV有机会与CD30阴性肿瘤细胞最终结合发挥杀伤作用。共聚焦显微镜和成像质谱流式细胞术研究均发现BV的结合以及摄取依赖于CD30阳性EVs，因此只有在CD30阳性EVs存在的情况下，BV才会对CD30阴性肿瘤细胞进行杀伤，为BV对CD30阴性肿瘤也能产生作用提供了解释，但仍需更多研究进行确认。

目前，美国食品药品监督管理局（FDA）已批准BV用于初治及复发难治HL、初治系统性间变性大细胞淋巴瘤（sALCL）或CD30阳性外周T细胞淋巴瘤（PTCL）、复发难治sALCL、原发皮肤间变性大细胞淋巴瘤（pcALCL）以及CD30阳性蕈样肉芽肿（MF）。2020年8月，BV通过中国国家药品监督管理局（NPMA）优先审评，正式获批用于CD30阳性的复发或难治性sALCL和经典型HL（cHL）。2021年4月，获批用于CD30阳性的既往接受过系统性治疗的pcALCL或MF。目前BV已在超过70个国家和地区获批用于治疗淋巴瘤。

三、维布妥昔单抗治疗CD30阳性淋巴瘤的关键性临床研究

1. HL：

（1）一线治疗：ECHELON-1研究经过5年长期随访，确立了BV联合化疗在未经治疗的晚期HL患者中的一线治疗地位。研究共入组1 334例患者，患者被1∶1随机分配到A-AVD（BV+阿霉素+长春花碱+达卡巴嗪）和ABVD组（博来霉素+阿霉素+长春花碱+达卡巴嗪）。中位随访60.9个月，A-AVD组和ABVD组的5年无进展生存（PFS）率分别为82.2％和75.3％。在2个疗程后PET阴性亚组人群中同样观察到患者从A-AVD方案中显著获益，A-AVD组和ABVD组的5年PFS率分别为84.9％和78.9％。安全性方面，两组周围神经病变发生率相似，A-AVD组和ABVD组分别为85％和86％，且能够随着时间的延长持续改善或缓解，A-AVD组持续性周围神经病变患者比例略高（19％对9％）[16]。

（2）复发难治：对于高危的复发/难治性HL患者，挽救治疗后行自体造血干细胞移植是标准治疗。BV具有较好的抗肿瘤作用，使患者有机会接受治愈性造血干细胞移植治疗。Walewski等[17]开展了一项全球多中心前瞻性、单臂Ⅳ期研究，评估在不适合行造血干细胞移植或多药化疗的复发/难治性HL中应用BV单药的疗效和安全性。研究共纳入60例患者，研究结果显示总缓解率（ORR）为50％，其中完全缓解率（CRR）12％，中位PFS和总生存（OS）时间分别为4.8个月和未达到，47％患者最终行造血干细胞移植。最常见的不良事件为周围神经病变（35％）和发热（18％），未见新的安全性信号。

此外，有研究首次发现BV联合纳武单抗是复发/难治HL患者安全且有效的挽救治疗方案[18]。研究共纳入91例患者，ORR和CRR分别为85％和67％，中位随访34.3个月，预估3年PFS率和OS率分别为77％和93％，进行自体造血干细胞移植巩固的患者预估3年PFS率可达91％，16例（18％）患者出现免疫相关不良事件（AE），使用系统性激素治疗后缓解。

（3）移植后维持治疗：AETHERA研究是一项国际多中心、安慰剂对照Ⅲ期研究，HL患者经过自体造血干细胞移植后存在疾病进展或复发高危因素则随机进入BV组和安慰剂组，研究共纳入329例患者。经过5年长期随访，发现BV能够为患者带来持续获益。BV组5年PFS率高于安慰剂组（59％对41％）。同样地，存在≥2个高危因素患者中BV组5年PFS率显著提高。移植后BV维持治疗能够显著延长至下一次治疗时间，是持续疾病控制的预测因子。虽然周围神经毒性是接受BV治疗患者中最常见的不良反应，但90％患者接受对症治疗后能够改善或恢复[19]。

BV在HL中的治疗地位确切，多项研究探索了BV联合方案应用于初治或复发/难治性HL。目前在HL中正在进行的含BV方案临床研究有10余项，包括BV联合化疗方案或免疫检查点抑制剂，以及BV维持与移植对比，或移植后BV维持在HL中的作用（表2）。

**表2:** 正在进行的维布妥昔单抗治疗霍奇金淋巴瘤的临床研究

NCT号	分期	研究设计	样本量	干预方案	患者类型	主要终点
NCT05243693	Ⅱ	单臂，开放性	30	BV-DHAP	复发难治	CRR
NCT04561206	Ⅱ	单臂，开放性	31	BV+纳武单抗	复发难治	PFS
NCT04378647	Ⅱ	随机对照，开放性	150	ESHAP±BV诱导，BV维持	复发难治	CRR（PET-CT 1～2分）
NCT03712202	Ⅱ	随机对照，开放性	264	1组A臂：AN；1组B臂：ABVD+纳武单抗；2组：AN-AVD	初发	PFS
NCT03652441	Ⅱ	单臂，开放性	21	BV维持	移植后维持	累积复发率
NCT03646123	Ⅱ	非随机，对照，开放性	240	A部分：A-AVD；B和C部分：AN-AD	初发	A部分：安全性；B和C部分：CRR
NCT03540849	Ⅱ	单臂，开放性	58	BV维持	移植后维持	累积复发率
NCT03474133	Ⅱ	单臂，开放性	80	BV单药	复发难治	持续CRR
NCT03233347	Ⅱ	单臂，开放性	82	A-AVD诱导，纳武单抗±BV维持	初发	PFS
NCT02661503	Ⅲ	随机对照，开放性	1 500	BrECADD对BEACOPP	初发	PFS
NCT01896999	Ⅰ/Ⅱ	随机对照，开放性	146	BV+伊匹单抗；BV+纳武单抗；BV+伊匹单抗+纳武单抗	复发难治	Ⅰ期：安全性；Ⅱ期：CRR

注 BV：维布妥昔单抗；DHAP：地塞米松+顺铂+阿糖胞苷；ESHAP：依托泊苷+甲泼尼龙+阿糖胞苷+顺铂；AN：维布妥昔单抗+纳武单抗；ABVD：阿霉素+博来霉素+长春花碱+达卡巴嗪；AVD：阿霉素+长春花碱+达卡巴嗪；A-AVD：维布妥昔单抗+阿霉素+长春花碱+达卡巴嗪；AD：阿霉素+达卡巴嗪；BrECADD：维布妥昔单抗+依托泊苷+阿霉素+环磷酰胺+达卡巴嗪+地塞米松；BEACOPP：博来霉素+依托泊苷+阿霉素+环磷酰胺+长春新碱+甲基苄肼+泼尼松；CRR：完全缓解率；PFS：无进展生存

2. PTCL：

（1）一线治疗：ECHELON-2研究是一项双模拟、随机双盲、安慰剂对照Ⅲ期研究，经过5年长期随访，确立了BV联合化疗在CD30阳性PTCL中的一线治疗地位。研究共纳入452例患者，1∶1随机分配到A+CHP组（BV+环磷酰胺+阿霉素+泼尼松）和CHOP组（环磷酰胺+阿霉素+长春新碱+泼尼松）。结果发现，A+CHP组的中位PFS时间明显较CHOP组延长（48.2个月对20.8个月，*P*＝0.011）。sALCL亚组能从A+CHP方案中显著获益。不良事件包括发热性中性粒细胞减少和周围神经病变。5年探索性亚组分析进一步证实A+CHP在各个亚组中具有一致性优势[20]。

（2）复发难治：一项关键性Ⅱ期研究确定了BV单药在复发/难治性sALCL中的地位。研究共纳入58例患者，中位随访71.4个月，随访超过40个月后未观察到疾病进展发生，ORR为86％，其中CRR为57％，5年PFS率和OS率分别为39％和60％。在38例获得完全缓解（CR）的患者中，16例接受造血干细胞移植巩固（其中自体和异基因造血干细胞移植各8例），这部分患者中位PFS和OS时间均未达到。安全性方面，周围神经系统毒性仍最常见（57％），但通过对症支持治疗，90％以上患者能够缓解或改善[21]。

ALCANZA研究是一项全球多中心、随机开放性Ⅲ期研究，证实了在既往接受过治疗的CD30阳性MF和pcALCL中，BV单药的疗效较研究者选择的常规疗法有优势。研究共纳入128例患者，1∶1随机分配至BV组和研究者选择的常规治疗组。中位随访45.9个月，BV组和常规治疗组中客观缓解持续≥4个月的患者比例分别为54.7％和12.5％，CRR为17.2％和1.6％，中位PFS时间为16.7个月和3.5个月。安全性方面，BV组周围神经病变发生率较高（69％）[22]。

近5年来，越来越多的研究结果显示，在非sALCL的复发/难治性CD30阳性PTCL和某些特定亚型如LyP及肠病相关T细胞淋巴瘤中，BV单药或联合化疗同样具有抗肿瘤活性[23]–[25]。目前尚有10余项正在进行的研究继续探索BV应用于移植前预处理，或联合免疫检查点抑制剂等方案应用于CD30阳性PTCL（表3）。

**表3:** 正在进行的维布妥昔单抗治疗CD30阳性PTCL的临床研究

NCT号	适应证	分期	研究设计	样本量	干预方案	患者类型	主要终点
NCT04837222	CD30阳性淋巴瘤	Ⅳ	非干预性	1 000	非干预性	初发，复发难治	安全性
NCT04569032	CD30阳性PTCL（表达<10%）	Ⅱ	双队列，开放性，非随机	80	BV-CHP	初发	ORR
NCT04334174	CD30阳性PTCL	Ⅱ	单臂，开放性	36	BV单药	移植后维持	安全性
NCT03587844	MF、LyP、SS	Ⅱ	序贯，开放性，非随机	58	BV单药	初发	ORR
NCT03947255	CD30阳性PTCL	Ⅱ	单臂，开放性	80	BV再治疗	BV后复发难治	ORR
NCT03409432	CD30阳性PTCL	Ⅱ	单臂，开放性	37	BV+来那度胺	复发难治	ORR
NCT03302728	PTCL	Ⅰ	单臂，开放性	24	BV+来那度胺	复发难治	安全性
NCT03264131	CD30阳性ATL	Ⅱ	单臂，开放性	28	BV-CHEP	初发，经治（最多1个疗程化疗）	CRR
NCT03246750	结外NK/T细胞淋巴瘤	Ⅰ/Ⅱ	单臂，开放性	36	B-MAD	初发	安全性
NCT03217643	CD30阳性肠病T细胞淋巴瘤Ⅰ型	Ⅱ	单臂，开放性	25	BV-CHP	初发	PFS
NCT03187210	CD30阳性PTCL	Ⅰ/Ⅱ	Ⅰ期：单臂，开放性；Ⅱ期：随机对照，开放性	60	BV-BeEAM对BeEAM	预处理	Ⅰ期：MTD；Ⅱ期：DFS
NCT02616965	皮肤T细胞淋巴瘤	Ⅰ	单臂，开放性	27	BV+罗米地辛	初发，复发难治	MTD，DLT
NCT02588651	CD30阳性PTCL（表达<10%）	Ⅱ	单臂，开放性	31	BV单药	复发难治	ORR
NCT01716806	CD30阳性PTCL或HL	Ⅱ	随机对照，开放性	180	BV单药（PTCL部分）	初发	ORR
NCT01703949	CD30阳性淋巴瘤	Ⅱ	单臂，开放性	40	BV单药，BV+纳武利尤单抗	复发难治	ORR

注 PTCL：外周T细胞淋巴瘤；MF：蕈样肉芽肿；LyP：淋巴瘤样丘疹病；SS：Sezary综合征；ATL：成人T细胞白血病/淋巴瘤；HL：霍奇金淋巴瘤；BV：维布妥昔单抗；CHP：环磷酰胺+阿霉素+泼尼松；CHEP：环磷酰胺+阿霉素+依托泊苷+泼尼松；B-MAD：维布妥昔单抗+甲氨蝶呤+左旋门冬酰胺酶+地塞米松；BeEAM：苯达莫司汀+依托泊苷+阿糖胞苷+美法仑；ORR：总缓解率；CRR：完全缓解率；PFS：无进展生存；MTD：最大耐受剂量；DFS：无疾病生存；DLT：剂量限制性毒性

3. DLBCL及其他B细胞淋巴瘤：

（1）一线治疗：Svoboda等[26]曾探索BV-R-CHP（BV+利妥昔单抗+环磷酰胺+阿霉素+泼尼松）方案一线治疗CD30阳性B细胞淋巴瘤的疗效和安全性。这项单臂Ⅰ/Ⅱ期研究安全性分析共纳入31例患者，研究结果显示ORR为100％，其中86％患者获得CR。值得注意的是，研究中5例DLBCL患者均取得较理想的疗效，截至文章发表，均未发生疾病进展。另有一项随机对照Ⅱ期研究试图比较R-CHOP与BV-R-CHP方案一线治疗DLBCL的疗效及安全性，但随着DLBCL一线治疗格局的变化，研究于2017年暂停。因此，BV联合方案一线治疗DLBCL的临床证据有待更多数据支持。

（2）复发难治：一项开放性Ⅱ期研究评估了BV用于复发/难治性CD30阳性非霍奇金淋巴瘤（NHL）的疗效，研究发现BV对不同CD30表达程度的复发/难治DLBCL均有效。其中49例DLBCL患者的ORR为44％，CRR为17％，缓解率与CD30表达情况无明显相关性。这些DLBCL患者中分别有76％和82％对一线治疗及末线治疗耐药，这部分人群ORR为44％（其中CRR 15％）。6例灰区淋巴瘤患者中1例CR，2例部分缓解。6例原发纵隔大B细胞淋巴瘤患者中1例CR，3例移植后淋巴细胞增殖性疾病患者中1例CR。安全性与已知风险一致[8]。

一项Ⅰ期/剂量拓展研究同样证实BV联合来那度胺是复发/难治性DLBCL患者的潜在治疗选择，ORR为57％，CRR为36％，中位PFS和OS时间分别为10.2个月和14.3个月[27]。基于这项研究结果开展的ECHELON-3研究是一项随机双盲、安慰剂对照、Ⅲ期研究，旨在评价BV对比安慰剂联合R2（利妥昔单抗+来那度胺）方案治疗复发/难治性DLBCL的疗效及安全性。上述研究及其他含BV方案治疗CD30阳性B细胞淋巴瘤的临床研究正在进行中（表4）。

**表4:** 正在进行的维布妥昔单抗治疗CD30阳性B细胞淋巴瘤的临床研究

NCT号	适应证	分期	研究设计	样本量	干预方案	患者类型	主要终点
NCT04745949	PMBCL	Ⅱ	单臂，开放性	40	BV+R-CHP+纳武单抗	初发	CRR
NCT04587687	FL	Ⅱ	单臂，开放性	23	BV+苯达莫司汀	复发难治	CRR，BOR
NCT04404283	DLBCL	Ⅲ	随机对照，双盲	400	BV；安慰剂+R2	复发难治	PFS
NCT03356054	DLBCL	Ⅰ/Ⅱ	单臂，开放性	37	BV-R-DHAP	复发难治	Ⅰ期：安全性；Ⅱ期：代谢CRR

注 PMBCL：原发纵隔大B细胞淋巴瘤；FL：滤泡性淋巴瘤；DLBCL：弥漫大B细胞淋巴瘤；BV：维布妥昔单抗；R-CHP：利妥昔单抗+环磷酰胺+阿霉素+泼尼松；R2：来那度胺+利妥昔单抗；R-DHAP：利妥昔单抗+地塞米松+顺铂+阿糖胞苷；CRR：完全缓解率；BOR：最佳缓解率；PFS：无进展生存

四、争议性问题

1. CD30表达检测方法尚不统一：临床上CD30表达状态是决定是否应用靶向CD30治疗药物的重要评估因素。CD30免疫组化蛋白表达和mRNA水平具有高度一致性，是临床实践中用于评价CD30表达的有力工具[2]。然而，不同的免疫组化技术、标本处理方法、抗体选择和CD30阳性界值均可能导致不同的CD30检测结果。目前比较常用的CD30检测试剂包括BerH2、1G12、JCM182等抗体。评估CD30表达率应提供肿瘤细胞表达情况，这对于大细胞淋巴瘤如ALCL并不困难，阳性率可用CD30阳性瘤细胞数/肿瘤细胞数来表示。但当肿瘤细胞较小或中等大小时，如AITL等，如何区分肿瘤细胞和背景细胞变得具有挑战性。在这种情况下，可用CD30阳性细胞数/视野细胞数判读CD30阳性率[28]。由于靶向药物的广泛使用，CD30表达检测在淋巴瘤的诊断、治疗、预后评价中被广泛应用，如何统一CD30检测方法和标准至关重要。在目前尚未统一的情况下，建议尽可能提供肿瘤细胞表达比例或整体淋巴细胞CD30表达比例，这是提供有效信息非常重要的方法。

2. CD30在淋巴瘤中的预后意义存在争议：

（1）DLBCL：有研究发现CD30阳性DLBCL更易出现LDH升高，IPI低危比例低，组织病理学上与非生发中心（non-GCB）亚型和EB病毒编码的RNA（EBER）阳性相关，是不良因素[11]。而Hu等[29]的研究结果却认为CD30阳性患者的OS和PFS率显著优于CD30阴性患者（5年OS率79％对59％，*P*＝0.001；5年PFS率73％对57％，*P*＝0.003）。一项荟萃分析结果也提示，无论用5％还是20％作为CD30阳性表达的界值，其在DLBCL中均与更好的预后相关[30]。

（2）PTCL：国际T细胞淋巴瘤工作项目对490例PTCL患者进行亚组分析未发现CD30具有预后提示意义，研究中大部分患者使用CHOP样方案，在除ALCL以外的其他病理亚型中，CD30阳性患者在CRR、5年PFS率和5年OS率上与CD30阴性患者相似[6]。Bisig等[31]发现CD30阳性PTCL-NOS患者与ALK阴性ALCL具有相似的分子学特征。而与CD30阳性PTCL-NOS及ALK阳性ALCL患者相比，CD30阴性PTCL-NOS患者中位PFS和OS时间更短，但由于ALK阳性ALCL本身预后较好，故该研究的CD30预后意义可能受此影响。NK/T细胞淋巴瘤中，多个研究发现CD30阳性表达与预后不良相关，CD30阳性NK/T细胞淋巴瘤患者的OS和PFS率均明显低于CD30阴性组[3]–[4]。

3. CD30表达与BV疗效相关性存在争议：目前无确切研究表明BV疗效与CD30表达相关。ALCANZA研究亚组研究中，根据≥2次皮肤活检中CD30表达最低水平（CD30_min_），将患者分为CD30_min_<10％的低表达组和CD30_min_≥10％的高表达组，研究结果发现两组都能从BV治疗中获益[32]。在多个复发难治CD30阳性淋巴瘤中，BV在任何CD30阳性水平均可见疗效，并且未观察到疗效和不同CD30表达水平有一致性[33]。

五、其他CD30靶点治疗药物

1. 抗体药物偶联物：其他目前正在临床研发中的靶向CD30-ADC药物主要是上海复旦张江/交联的F0002-ADC。F0002是一种注射用重组人鼠嵌合抗CD30单克隆抗体-硫醚连接子（MCC）-细胞毒药物DM1偶联剂，与BV相比，F0002采不具备“旁观者效应”，仅特异性杀伤肿瘤细胞[34]。目前F0002-ADC在国内开展的复发/难治性CD30阳性血液肿瘤Ⅰ期临床研究（CTR20190341）和复发/难治性CD30阳性外周T细胞淋巴瘤Ⅰb期临床研究（CTR20211977）正在招募中。

2. 双特异性抗体：目前尚无靶向CD30双特异性抗体在临床获批，由Affimed公司研发的AFM13（CD30/CD16A）是一种创新型固有免疫细胞激动剂。AFM13通过特异性结合肿瘤细胞表达的CD30和NK细胞、巨噬细胞表达的CD16A，激活固有免疫细胞而发挥抗肿瘤效应，是一种固有免疫系统激活的新途径。已有研究证实AFM13在多线治疗后复发难治CD30阳性皮肤淋巴瘤中的ORR可达到40％，且BV治疗失败后患者仍可从中获益[35]。另外，尚有一项评估AFM13单药治疗复发难治CD30阳性PTCL或转化性MF的Ⅱ期注册性临床研究（NCT04101331）正在进行中。

3. 嵌合抗体受体T（CAR-T）细胞：由于BV在HL及ALCL中的成功尝试，靶向CD30 CAR-T细胞治疗有望成为CD30阳性淋巴瘤新的治疗选择。在一项CD30 CAR-T细胞治疗复发难治CD30阳性淋巴瘤的Ⅰ期研究中，7例HL患者中2例获得CR，2例ALCL患者中有1例CR并持续缓解9个月，且具有良好的安全性[36]。国内首个被批准进行临床研究的CD30 CAR-T细胞产品（BRD-01）研究结果也证实在9例CD30阳性复发HL及ALCL患者中7例达到CR，且其中3例CR患者能够获得超过2年的长期生存。总体研究人群发生细胞因子风暴（CRS）的比例为66.7％，大部分为1～2级[37]。

六、BV治疗中国CD30阳性淋巴瘤患者的真实世界研究（BRAVE研究）

国内与BV治疗相关的真实世界数据非常有限。本中心牵头开展了一项研究BV在中国CD30阳性淋巴瘤患者中使用的安全性和有效性的前瞻性、观察性、非干预性、多中心真实世界临床实践研究（NCT04837222），研究将纳入连续1 000例接受BV治疗的CD30阳性淋巴瘤患者。关键入组标准包括：接受BV治疗或即将接受BV治疗的成年患者；患者/法定监护人必须能够阅读、理解并签署知情同意书；由研究中心检测CD30阳性的淋巴瘤患者（任何CD30表达）。排除标准包括目前参加或计划参加任何干预性临床试验的患者，以及研究者认为有其他原因使该患者不适合参加本研究。由于CD30阳性淋巴瘤不是常见疾病，且药物可及性有限，因此1 000例CD30阳性淋巴瘤患者将会从30家血液病中心招募。研究者将根据常规诊疗经验确定治疗方案以及实验室和临床评估的频率。所有患者将按照常规临床实践进行随访，数据将在3、6、9、12、18、24个月记录，除非患者撤回知情同意书、死亡或失访，按照优选顺序。通过与入组患者的积极联系最大限度地减少随访损失，确保所有临床相关的结果都被记录。

研究主要终点为严重不良反应事件，次要终点包括不良事件、药物不良反应、剂量调整、接受BV的患者特征、BV的应用、应用BV的周期数、疾病特征、至下次治疗的时间、总体反应率、反应持续时间、PFS率、OS率、生活质量和药物经济学。该研究旨在及时获得有关BV在中国CD30阳性淋巴瘤患者中的安全性和有效性的真实世界数据。

七、总结

CD30在淋巴瘤中广泛表达，其生物学意义和临床意义仍然有待探索，临床试验已证明BV治疗多种CD30阳性淋巴瘤的有效性和安全性。目前国内与BV治疗相关的真实世界数据非常有限，我们期望通过中国人群BV真实世界研究（BRAVE研究）数据及时获得更多安全性和有效性数据，为BV获益人群、生物标志物、与CD30表达水平相关性等关键信息提供更多线索。

## References

[1] Dürkop H, Latza U, Hummel M (1992). Molecular cloning and expression of a new member of the nerve growth factor receptor family that is characteristic for Hodgkin's disease[J]. Cell.

[2] Bossard C, Dobay MP, Parrens M (2014). Immunohistochemistry as a valuable tool to assess CD30 expression in peripheral T-cell lymphomas: high correlation with mRNA levels[J]. Blood.

[3] Sabattini E, Pizzi M, Tabanelli V (2013). CD30 expression in peripheral T-cell lymphomas[J]. Haematologica.

[4] Federico M, Bellei M, Luminari S (2015). CD30+ expression in Peripheral T-cell lymphomas (PTCLs): A subset analysis from the international, prospective T-Cell Project[J]. J Clin Oncol.

[5] Li P, Jiang L, Zhang X (2014). CD30 expression is a novel prognostic indicator in extranodal natural killer/T-cell lymphoma, nasal type[J]. BMC Cancer.

[6] Wang GN, Zhao WG, Li L (2017). Prognostic significance of CD30 expression in nasal natural killer/T-cell lymphoma[J]. Oncol Lett.

[7] Slack GW, Steidl C, Sehn LH (2014). CD30 expression in de novo diffuse large B-cell lymphoma: a population-based study from British Columbia[J]. Br J Haematol.

[8] Jacobsen ED, Sharman JP, Oki Y (2015). Brentuximab vedotin demonstrates objective responses in a phase 2 study of relapsed/refractory DLBCL with variable CD30 expression[J]. Blood.

[9] Huo YJ, Xu PP, Fu D (2022). Molecular heterogeneity of CD30+ diffuse large B-cell lymphoma with prognostic significance and therapeutic implication[J]. Blood Cancer J.

[10] Malysz J, Erdman P, Klapper J (2016). Clinical Implications of CD30 Expression in Aggressive B-Cell Lymphomas[J]. Clin Lymphoma Myeloma Leuk.

[11] Hoeller S, Zihler D, Zlobec I (2010). BOB.1, CD79a and cyclin E are the most appropriate markers to discriminate classical Hodgkin's lymphoma from primary mediastinal large B-cell lymphoma[J]. Histopathology.

[12] Higgins JP, Warnke RA (1999). CD30 expression is common in mediastinal large B-cell lymphoma[J]. Am J Clin Pathol.

[13] Gardner LJ, Polski JM, Evans HL (2001). CD30 expression in follicular lymphoma[J]. Arch Pathol Lab Med.

[14] Deng C, Pan B, O'Connor OA (2013). Brentuximab vedotin[J]. Clin Cancer Res.

[15] Lobastova L, Lettau M, Babatz F (2021). CD30-Positive Extracellular Vesicles Enable the Targeting of CD30-Negative DLBCL Cells by the CD30 Antibody-Drug Conjugate Brentuximab Vedotin[J]. Front Cell Dev Biol.

[16] Straus DJ, Długosz-Danecka M, Connors JM (2021). Brentuximab vedotin with chemotherapy for stage III or IV classical Hodgkin lymphoma (ECHELON-1): 5-year update of an international, open-label, randomised, phase 3 trial[J]. Lancet Haematol.

[17] Walewski J, Hellmann A, Siritanaratkul N (2018). Prospective study of brentuximab vedotin in relapsed/refractory Hodgkin lymphoma patients who are not suitable for stem cell transplant or multi-agent chemotherapy[J]. Br J Haematol.

[18] Advani RH, Moskowitz AJ, Bartlett NL (2021). Brentuximab vedotin in combination with nivolumab in relapsed or refractory Hodgkin lymphoma: 3-year study results[J]. Blood.

[19] Moskowitz CH, Walewski J, Nademanee A (2018). Five-year PFS from the AETHERA trial of brentuximab vedotin for Hodgkin lymphoma at high risk of progression or relapse[J]. Blood.

[20] Horwitz SM, Savage KJ, Illidge T (2021). The Echelon-2 Trial: 5-Year Exploratory Subgroup Analyses of a Randomized, Double-Blind, Phase 3 Study of Brentuximab Vedotin and CHP (A+CHP) Vs CHOP in Frontline Treatment of Pts with CD30-Positive Peripheral T-Cell Lymphoma[J]. Blood.

[21] Pro B, Advani R, Brice P (2017). Five-year results of brentuximab vedotin in patients with relapsed or refractory systemic anaplastic large cell lymphoma[J]. Blood.

[22] Horwitz SM, Scarisbrick JJ, Dummer R (2021). Randomized phase 3 ALCANZA study of brentuximab vedotin vs physician's choice in cutaneous T-cell lymphoma: final data[J]. Blood Adv.

[23] Kim M, Lee JO, Koh J (2021). A phase II study of brentuximab vedotin in patients with relapsed or refractory Epstein-Barr virus-positive and CD30-positive lymphomas[J]. Haematologica.

[24] Kim SJ, Yoon DH, Kim JS (2020). Efficacy of Brentuximab Vedotin in Relapsed or Refractory High-CD30-Expressing Non-Hodgkin Lymphomas: Results of a Multicenter, Open-Labeled Phase II Trial[J]. Cancer Res Treat.

[25] Sibon D, Khater S, Bruneau J (2021). The Eatl-001 Trial: Results of a Phase 2 Study of Brentuximab Vedotin and CHP Followed By Consolidation with High-Dose Therapy - Autologous Stem-Cell Transplantation (HDT-ASCT) in the Frontline Treatment of Patients with Enteropathy-Associated T-Cell Lymphoma[J]. Blood.

[26] Svoboda J, Bair SM, Landsburg DJ (2021). Brentuximab vedotin in combination with rituximab, cyclophosphamide, doxorubicin, and prednisone as frontline treatment for patients with CD30-positive B-cell lymphomas[J]. Haematologica.

[27] Ward JP, Berrien-Elliott MM, Gomez F (2022). Phase 1/dose expansion trial of brentuximab vedotin and lenalidomide in relapsed or refractory diffuse large B-cell lymphoma[J]. Blood.

[28] Horwitz S, O'Connor OA, Pro B (2019). Brentuximab vedotin with chemotherapy for CD30-positive peripheral T-cell lymphoma (ECHELON-2): a global, double-blind, randomised, phase 3 trial[J]. Lancet.

[29] Hu S, Xu-Monette ZY, Balasubramanyam A (2013). CD30 expression defines a novel subgroup of diffuse large B-cell lymphoma with favorable prognosis and distinct gene expression signature: a report from the International DLBCL Rituximab-CHOP Consortium Program Study[J]. Blood.

[30] Rodrigues-Fernandes CI, Abreu LG, Radhakrishnan R (2021). Prognostic significance of CD30 expression in diffuse large B-cell lymphoma: A systematic review with meta-analysis[J]. J Oral Pathol Med.

[31] Bisig B, de Reyniès A, Bonnet C (2013). CD30-positive peripheral T-cell lymphomas share molecular and phenotypic features[J]. Haematologica.

[32] Kim YH, Prince HM, Whittaker S (2021). Response to brentuximab vedotin versus physician's choice by CD30 expression and large cell transformation status in patients with mycosis fungoides: An ALCANZA sub-analysis[J]. Eur J Cancer.

[33] Jagadeesh D, Horwitz SM, Bartlett NL (2019). Response to brentuximab vedotin by CD30 expression: Results from five trials in PTCL, CTCL, and B-cell lymphomas[J]. J Clin Oncol.

[34] Shen Y, Yang T, Cao X (2019). Conjugation of DM1 to anti-CD30 antibody has potential antitumor activity in CD30-positive hematological malignancies with lower systemic toxicity[J]. MAbs.

[35] Sawas A, Chen PH, Lipschitz M (2020). Clinical and Biological Evaluation of the Novel CD30/CD16A Tetravalent Bispecific Antibody (AFM13) in Relapsed or Refractory CD30-Positive Lymphoma with Cutaneous Presentation: A Biomarker Phase Ib/IIa Study[J]. Blood.

[36] Ramos CA, Ballard B, Zhang H (2017). Clinical and immunological responses after CD30-specific chimeric antigen receptor-redirected lymphocytes[J]. J Clin Invest.

[37] Wang D, Zeng C, Xu B (2020). Anti-CD30 chimeric antigen receptor T cell therapy for relapsed/refractory CD30(+) lymphoma patients[J]. Blood Cancer J.

